# Vitamin D Induces Increased Systolic Arterial Pressure via Vascular Reactivity and Mechanical Properties

**DOI:** 10.1371/journal.pone.0098895

**Published:** 2014-06-12

**Authors:** Priscila Portugal dos Santos, Bruna Paola Murino Rafacho, Andréa de Freitas Gonçalves, Rodrigo Gibin Jaldin, Thiago Bruder do Nascimento, Marcondes Alves Barbosa Silva, Stêfany Bruno Assis Cau, Meliza Goi Roscani, Paula Schimdt Azevedo, Marcos Ferreira Minicucci, Rita de Cássia Tostes, Leonardo Antonio Memede Zornoff, Sergio Alberto Rupp de Paiva

**Affiliations:** 1 Department of Internal Medicine, Botucatu Medical School, UNESP - Universidade Estadual Paulista, Botucatu, São Paulo, Brazil; 2 Department of Surgery and Orthopaedics, Botucatu Medical School, UNESP - Universidade Estadual Paulista, Botucatu, São Paulo, Brazil; 3 Department of Pharmacology, School of Medicine at Ribeirao Preto - USP, University of São Paulo, São Paulo, Brazil; University of São Paulo School of Medicine, Brazil

## Abstract

**Background/Aims:**

The aim of this study was to evaluate whether supplementation of high doses of cholecalciferol for two months in normotensive rats results in increased systolic arterial pressure and which are the mechanisms involved. Specifically, this study assesses the potential effect on cardiac output as well as the changes in aortic structure and functional properties.

**Methods:**

Male Wistar rats were divided into three groups: 1) Control group (C, n = 20), with no supplementation of vitamin D, 2) VD3 (n = 19), supplemented with 3,000 IU vitamin D/kg of chow; 3) VD10 (n = 21), supplemented with 10,000 IU vitamin D/kg of chow. After two months, echocardiographic analyses, measurements of systolic arterial pressure (SAP), vascular reactivity, reactive oxygen species (ROS) generation, mechanical properties, histological analysis and metalloproteinase-2 and -9 activity were performed.

**Results:**

SAP was higher in VD3 and VD10 than in C rats (p = 0.001). Echocardiographic variables were not different among groups. Responses to phenylephrine in endothelium-denuded aortas was higher in VD3 compared to the C group (p = 0.041). Vascular relaxation induced by acetylcholine (p = 0.023) and sodium nitroprusside (p = 0.005) was impaired in both supplemented groups compared to the C group and apocynin treatment reversed impaired vasodilation. Collagen volume fraction (<0.001) and MMP-2 activity (p = 0.025) was higher in VD10 group compared to the VD3 group. Elastin volume fraction was lower in VD10 than in C and yield point was lower in VD3 than in C.

**Conclusion:**

Our findings support the view that vitamin D supplementation increases arterial pressure in normotensive rats and this is associated with structural and functional vascular changes, modulated by NADPH oxidase, nitric oxide, and extracellular matrix components.

## Introduction

Vitamin D (VD) is a fat-soluble compound primarily obtained through cutaneous synthesis. The remainder of VD may be obtained from supplements or foods, but few foods contain substantial amounts of VD [1], [2].

The prevalence of VD deficiency has increased in recent years [3], becoming a public health problem worldwide [4]. It is estimated that one billion people worldwide are either VD insufficient or deficient [3]. Furthermore, VD deficiency is associated with an increased risk of developing several chronic diseases [2], [5], [6], [7], [8], [9], [10], [11]. Therefore, researchers have recommended increased sun exposure, food fortification and VD supplementation, both for people at higher risk for hypovitaminosis D and for the general population [12], [13], [14]. Medium and long term effects of VD supplementation with doses above 4,000 IU/day are not well known, and risks may not be disregarded, although toxic effects are rare [15]. VD dose of 4,000 IU/day was recently recommended by Institute of Medicine as Upper Level Intake [16].

The classic function of VD is to regulate calcium and phosphorus homeostasis, but VD also modulates the function of a variety of non-classical target tissues, including vascular smooth muscle cells (VSMC) and endothelial cells [17], [18], [19]. Several mechanisms have been proposed on how VD could be involved in blood pressure regulation and the pathophysiology of arterial hypertension. VD effects on the renin angiotensin aldosterone system (RAAS), by down-regulates renin expression, have been extensively investigated by experimental studies [20], [21]. Other mechanisms linking VD and blood pressure may be related to direct VD effects on the vasculature [22], [23], [24], [25].

Several randomized controlled trials (RCTs) evaluating the effects of VD supplementation on blood pressure have been conducted with inconsistent results [26], [27], [28], [29], [30], [31]. These results along with some other RCTs showing no significant blood pressure effect of VD in largely normotensive individuals [27], [32], [33], [34], suggest that if antihypertensive effects of VD are actually present, these may only be observed in groups with both low VD levels and high blood pressure [35].

On the other hand, in experimental studies Bukoski & Xue (1993) [36] and Haffner *et al*. (2005) [37] showed that administering 1,25-dihydroxyvitamin D increases systolic blood pressure in normotensive rats. In agreement with this result, our group has shown that normotensive rats supplemented for two months with cholecalciferol also presented with higher systolic blood pressure [38].

Systemic arterial blood pressure is determined by the cardiac output and systemic vascular resistance [39], [40]. Vascular resistance is set predominantly by the vascular tone in the arterial tree but is also influenced by alterations in the vascular structural, functional and mechanical properties. Structural and functional abnormalities in the vasculature may be due to endothelial dysfunction, increased vascular oxidative stress, vascular remodeling, and decreased compliance. These factors directly impact vascular resistance and may antedate hypertension and contribute to its pathogenesis. Therefore, endothelial dysfunction, increased oxidative stress, vascular remodeling and decreased compliance directly impact vascular resistance [40], [41]. Experimental studies with rats and cultured cells have shown that VD supplementation is associated with some of these vascular changes [22], [23], [24], [25]. However, the mechanisms through which VD increases systolic arterial pressure are unclear.

Therefore, we tested the hypothesis that increased systolic arterial pressure in normotensive rats after VD supplementation results from both an increase in cardiac output and vascular resistance.

The aim of this study was to evaluate whether supplementing high doses of cholecalciferol for two months in normotensive rats increases systolic arterial pressure and which are the mechanisms involved. Specifically, this study assesses the potential effect on cardiac output as well as the changes in aortic structure and functional properties.

## Materials and Methods

### Experimental protocol

All experiments and procedures were performed in accordance with the National Institute of Health's Guidelines for the Care and Use of Laboratory Animals and were approved by the Ethics Committee for Animal Experimentation of the Botucatu Medical School, UNESP, São Paulo, Brazil (2008/694). Male Wistar rats weighting 250 g were randomly allocated into three groups and fed a cereal-based chow diet for two months: 1) control group (C, n = 20), with no supplementation of VD (Cereal-based diet - Nuvilab CR1, with the approximate composition (kg mixture): protein, 220 g; fat, 40 g; mineral, 100 g; fiber, 80 g and VD, 1,800 IU); 2) VD3 (n = 19), supplemented with 3,000 IU VD/kg of chow; 3) VD10 (n = 21), supplemented with 10,000 IU VD/kg of chow. All animals were fed the same amount of chow. All animal groups received 10 ml of corn oil per kg of chow. Supplementation with VD was made by adding cholecalciferol (Sigma-Aldrich, MO, USA) diluted in the corn oil.

The National Research Council recommended to rats the amount of 1,000 IU of VD per kg of chow for rats [42]. However, they do not have defined an upper intake level. Therefore, we use the relation of ten times the recommended daily dose to have our high dose. Shepard & DeLuca (1980) showed that rats supplemented with doses above 1,000 IU of VD/day (∼ 30,000 IU/kg of chow) presented toxicity signs such as irritability, diarrhea, loss of appetite, decrease in weight gain, the kidneys became mottled and in their kidneys to take on a grayish-white color indicative of calcification [43]. The doses used in our study were 4.8 and 11.8 times higher than recommended dose for rats and did not reach the 1,000 IU/day considered toxic by Shepard & DeLuca (1980) [43]. Therefore, the doses used in the present study were considered non toxic.

After two months of VD supplementation, the rats were submitted to measurements of systolic arterial pressure (SAP) and echocardiographic analyses. Thus, the animals were euthanized, and the thoracic aortas from each animal was carefully removed, and the segments were used to analyze the vascular reactivity, assessment of vascular reactive species, mechanical proprieties, histological analysis and metalloproteinase-2 and -9 activity.

### Systolic arterial pressure

The systolic arterial pressure of the tail was measured one week before euthanasia with a tail plethysmograph. The animals were warmed in a wooden box at 40°C with heat generated by two incandescent lamps for four minutes to cause vasodilation artery tail and were then transferred to an iron cylindrical support that was specially designed to allow total exposure of the animal's tail. A sensor (KSM-microphone) was placed in the proximal region of the tail, coupled to an electro-sphygmomanometer, Narco Bio-System, model 709-0610 (International Biomedical Inc, TX, USA) [44]. The electro-sphygmomanometer was attached to a computer where the systolic arterial pressure was measured with the software Biopac Student Lab PRO (Biopac Systems Inc., CA, USA).

### Echocardiographic study

After 2 months, all animals were weighed and evaluated with a transthoracic echocardiographic exam [45]. The exams were performed using a commercially available echocardiographic machine (General Electric Medical Systems, Vivid S6, Tirat Carmel, Israel) equipped with 5–12 MHz phased array transducer. All measurements were obtained by the same observer according to the leading-edge method recommended by the American Society of Echocardiography/European Association of Echocardiography [46]. The data represent the mean of measurements from at least five consecutive cardiac cycles. The rats were lightly anaesthetized with an intramuscular injection of a solution composed of ketamine (50 mg/kg) plus xylazine (1 mg/kg). The rat chests were shaved, and the rats were placed in a left lateral position. Targeted 2-D M-mode echocardiograms were obtained from short-axis views of the left ventricle (LV) at or just below the tip of the mitral-valve leaflets and at the level of the aortic valve and left atrium. M-mode images of the LV and left atrium were recorded at a sweep speed of 100 mm/s. The LV end-diastolic dimension (LVEDD) was measured at maximal diastolic dimension. The left atrium was measured at its maximal diameter. The LV systolic function was assessed by calculating the ejection fraction [(LVEDD3 – LVESD3)/LVEDD3]. The transmitral diastolic flow (E and A) velocities were obtained from the apical four-chamber view. The E/A ratio was used as an index of the LV diastolic function.

### Vascular reactivity

The thoracic aorta was isolated and cleaned of connective tissue and fat. Aortic rings, 4 mm in length, were cut and mounted for isometric tension recording. The rings were placed in bath chambers (5 ml) for isolated organs (Mulvany Myograph) [47] containing modified Krebs salt solution of the following composition (mM): NaCl 130, CaCl_2_ 1.6, MgSO_4_ 1.2, KH_2_PO_4_ 1.2, KCl 4.7, NaHCO_3_ 14.9, glucose 5.5, which was maintained at 37°C, pH 7.4, and bubbled with 95% O_2_ and 5% CO_2_ [48]. The responses were recorded on a computer system using the Chart V4.04, PowerLab ADInstruments (2000) program. The aortic rings were submitted to a tension of 30 mN during a 45-min equilibration period. In some aortic rings, the endothelium was gently removed with a needle. After equilibration, rings were pharmacologically tested for endothelial integrity with 60 mmol/L KCl. The concentration-response curves to phenylephrine (Phe) (10^−10^ to 10^−5^ M) were obtained from the arteries with endothelium intact or denuded. To assess the endothelium dependent and endothelium-independent relaxations, aortic rings pre-contracted with Phe (10^−5^ M) were used to construct cumulative concentration-response curves to acetylcholine (Ach) (10^−11^ to 10^−5^ M) and sodium nitroprusside (NPS) (10^−11^ to 10^−6^ M). Relaxation was calculated as a percentage of the contraction induced by Phe (10^−5^ M). In addition, the involvement of reactive species NADPH-dependent and the contribution of nitric oxide (NO) to relaxation was assessed by the pre-incubation of apocynin (3×10^−4^ M), which was incubated 30 min before the relaxation induced by Ach and NPS.

### Assessment of 25-hydroxyvitamin D3 and calcium and phosphorus

Plasma concentrations of 25-hydroxycholecalciferol (25(OH)D3) were measured by high performance liquid chromatography (HPLC) as described by Asknes (1992) [49]. Extraction of 25(OH)D3 was performed with 500 µl plasma samples that were placed in disposable glass test tubes, and 500 µl of methanol isopropanol (90∶10 v/v) was added and the tubes were vortex mixed for 15 s. A 1.5-ml aliquot of n-hexane was added and the tubes vortex mixed for 60 s and centrifuged at 1000 g for 3 min. The n-hexane layer was carefully transferred to a tapered microvial for autosampler and evaporated to dryness with a stream of N_2_. The samples were redissolved in 125 µl of methanol and injected by an autosampling injector into a C-18 reverse phase column. The apparatus used was Waters 2695 chromatograph with photodiode-detector Waters 2996. The mobile phase consisted of a mixture of water and methanol (85∶15, v/v)) at a flow rate of 0.5 ml min-', followed by a 20-min. The detector wave length was set at 265 nm. 25(OH)D3 was quantified by determining peak areas on high-performance liquid chromatograms, calibrated against known amounts of standards (H4014 Sigma Co. USA).

Serum concentrations of calcium and phosphorus were measured in through arzenazo III method and colorimetric method, respectively (test kit Labor Lab, São Paulo, Brazil).

### Histological analysis

The first 5 mm of the aorta was cleaned of connective tissue and immediately fixed in 10% buffered formalin and embedded in paraffin. Five-micron-thick sections were stained with hematoxylin and eosin (HE), collagen-specific stain picrosirius red (Sirius red F3BA in aqueous saturated picric acid) and Calleja's stain to evaluate the elastin. The measurements were obtained from digital images that were collected with a video camera that was attached to a Leica microscope; the images were analyzed with the software Image-Pro Plus 3.0 (Media Cybernetics; Silver Spring, MD, USA). The media cross-sectional area (CSA) was calculated by subtracting the lumen internal area (Ai) from the external area (Ae), which was measured in the tissue sections (50x). The external diameter (ED) and the internal diameter (ID) were calculated as the square root of 4Ae/π and 4Ai/π, respectively. Media thickness (M) was calculated as (ED-ID)/2. Finally, M to lumen diameter (M/L) was also calculated [50].

The collagen volume fraction and elastin volume fraction was determined for the entire aortic section by analyzing the digital images that were captured under polarized light (400× magnification). These volume fractions were calculated as the sum of all connective tissue areas divided by the sum of all connective tissue and aorta areas. On average, 15 microscopic fields were analyzed per aorta [51].

### Mechanical properties analysis of the aorta

Aortic mechanical properties were studied in rat thoracic aortas. Before mechanical testing, 2-mm aorta fragments were promptly immersed in saline solution containing 0.25 mg/mL of papaverine to relax the muscle bundles of the arteries and to standardize the state of muscle tension in all aortic samples. The mechanical analysis was performed using a EMIC DL 10.000 Universal Machine of Mechanical Assays (Equipments and Testing Systems, Ltd., PR, Brazil). The aortas were immediately fixed with grasping clamps using smooth non-cutting metallic bars fastened with two screws. The stretching speed was 30 mm/minute and a 50-N load cell was used. Failure load, yield point (by Johnson's method), and stiffness were obtained. Failure load may be defined as the highest load tolerated by materials until rupture. Yield point is the maximum tension value below which materials comply with Hooke's law (in which the tension-deformation function is linear). Beyond the yield point, some degree of lesion may be found and plastic deformation materials may already be present, making the return to initial length impossible even if the loading stops. Stiffness is the linear and constant numeric relation between load and elongation calculated at the yield point [52].

### Metalloproteinase-2 and -9 activities by gelatin zymography

The metalloproteinase (MMP)-2 and -9 activities were determined, as reported previously [53]. Briefly, the aortic samples were homogenized in buffer containing 50 mM Tris, pH 7.4; 0.2 M NaCl, 0.1% Triton X and 10 mM CaCl2. The tissue extracts were subjected to electrophoresis on 8% SDS-polyacrylamide containing 1% gelatin. Electrophoresis was performed in a Bio-Rad apparatus at 100 V for 2 h at 4°C. After electrophoresis, the gel was incubated for 1 h at room temperature in 2.5% Triton-X-100, washed with 50 mM Tris pH 8.4 and incubated at 37°C for 20 hours in 50 mM Tris pH 8.4 containing 5 mM CaCl2. The staining was performed for 1 h with 0.5% coomassie blue and destaining in 30% methanol and 10% acetic acid until clear bands over a dark background were observed. The gels were photographed with an image analyzer (Carestream Molecular Imaging, Carestream, Inc., USA), and the optic density of each metalloproteinases band (measured in pixels) was quantified using Gel pro-3.1 software [54]. The inactive, pro and active forms of MMP-2 were identified as bands at 75, 72 and 64 kDa [55], respectively, and the pro and active MMP-9 were identified as band at 92 and 80 KDa, respectively [56].

### Data and statistical analysis

The concentration of Ach and NPS producing half-maximal relaxation (i.e., EC_50_) and the maximal relaxation of the NE contractile effect were estimated by linear regression analysis (fitted to the Hill equation) from the log concentration-response curves and expressed as -log EC_50_ (pD_2_ values) and as the percent of maximal relaxation. Between-group comparisons were made by a 1-way analysis of variance for variables with normal distribution. Otherwise, between-group comparisons were made using the Kruskal-Wallis test. The association between variables was assessed by Pearson correlation test. Data were expressed as the mean ± SD or medians (including the lower and upper quartiles). Data analysis was carried out with SigmaStat for Windows v3.5 (SPSS Inc., IL, USA). A significance level of 5% was used.

## Results

The daily intake of VD, plasma 25(OH)D3 and serum calcium and phosphorus are listed in Table 1. The daily intake of VD and serum calcium and phosphorus were higher in supplemented groups compared to control. Plasma 25(OH)D3 was higher in VD10 than control.

**Table 1:** Vitamin D and food ingestion, serum calcium and phosphorus and plasma 25 (OH) D_3_.

Variable	C	VD3	VD10	p
**Food ingestion (g/day)**	25.5±1.5 (20)	25.9±2.2 (19)	25.2±2.2 (21)	0.681
**Vitamin D ingestion (IU/day)**	45.3 (44.2–48.2) (20)	126.3 (116.7–128.5)^*^ (19)	309.2 (288.6–317.6)^*#^ (21)	<0.001
**25 (OH) D_3_ (ng/ml)**	15.0 (13.2–20.7) (5)	25.5 (19.0–40.5) (5)	37.0 (34.1–40.0)^*^ (5)	0.016
**Ca (mg/dl)**	8.24±0.36^a^ (20)	9.32±0.32^*^ (19)	9.44±0.15^*^ (21)	0.016
**P (mg/dl)**	5.90 (5.65–6.10)^a^ (20)	6.80 (6.35–8.00)^*^ (19)	7.60 (6.95–8.78)^*^ (21)	<0.001

Data are expressed as mean ± standard deviation of mean or median with 25 and 75 percentiles, numbers in parentheses indicate the numbers of animals included in each experimental group. C: control group (no supplementation with vitamin D); VD3: supplemented with 3,000 IU VD/kg of chow; VD10: supplemented with 10,000 IU VD/kg of chow; 25 (OH) D_3_: plasma 25-hydroxycholecalciferol; Ca: serum calcium; P: serum phosphorus. * p<0.05 versus control group; # p<0.05 versus VD3 group.

The data on systolic blood pressure and echocardiographic variables are listed in Table 2. Systolic arterial pressure was higher in both the supplemented groups compared to the control. The echocardiographic variables (i.e., heart rate, cardiac output, systolic and diastolic function and morphological variables) were not different when comparisons were performed among the three groups.

**Table 2:** Systolic arterial pressure, body weight and echocardiographic data.

Variable	C (n = 20)	VD3 (n = 19)	VD10 (n = 21)	p
**BW (g)**	402±33	411±38	405±23	0.700
**SAP (mmHg)**	119±7.9	127±9.2^*^	130±9.9^*^	**0.001**
**HR (bpm)**	318±37.8	322±37.9	315±42.4	0.845
**CO (ml/min)**	94±35	108±29	105±26	0.322
**EF**	0.88±0.08	0.90±0.04	0.92±0.04	0.119
**E/A**	1.48 (1.35–1.60)	1.49 (1.33–1.58)	1.48 (1.22–1.77)	0.992
**LA (mm)**	4.3±0.7	4.6±0.7	4.4±1.0	0.590
**LA/BW (mm/kg)**	10.1 (9.3–12.7)	11.0 (9.8–12.8)	9.9 (9.0–12.6)	0.515
**LVEDD (mm)**	7.0±0.7	7.2±0.6	7.1±0.5	0.475
**LVEDD/BW (mm/kg)**	17.2 (15.8–18.9)	17.0 (16.1–19.2)	17.5 (16.7–19.0)	0.857
**LVM (mg)**	621±149	695±115	698±128	0.119

Data are expressed as mean ± standard deviation of mean or median with 25 and 75 percentiles. n: number of rats. C: control group (no supplementation with vitamin D); VD3: supplemented with 3,000 IU VD/kg of chow; VD10: supplemented with 10,000 IU VD/kg of chow. BW: body weight; SAP: Systolic arterial pressure; HR: heart rate; CO: cardiac output; EF: ejection fraction; E: E wave; A: A wave; LA: left atrium; LVEDD: left ventricular (LV) end-diastolic diameter; LVM: left ventricular mass. * p<0.05 versus control group.

The blood pressure was not associated with serum calcium (r = 0.05 e p = 0.77).

Table 3 shows pharmacological parameters obtained from the cumulative concentration-response curves to Phe performed in endothelium-intact and endothelium-denuded aortas. Supplementation with 3,000 IU of VD significantly showed higher aortic Phe pD_2_ values in the endothelium-denuded aortas compared to the control group. No changes in the Phe maximal response were observed. However, changes in Phe responses were not observed in the endothelium-intact aortas.

**Table 3:** Summary of pD_2_ and Maximal Response values.

Variable		C	VD3	VD10	p
**Phe E^−^ (%KCl)**	**pD_2_**	7.9±0.8 (5)	8.8±0.6^*^ (6)	8.1±0.3 (6)	**0.041**
	**Maximal Response**	157.0±37.5 (5)	250.3±92.3 (6)	197.0±49.5 (6)	0.095
**Phe E^+^ (%KCl)**	**pD_2_**	7.4±0.4 (5)	7.7±0.2 (5)	7.3±0.4 (6)	0.236
	**Maximal Response**	148.7±41.9 (5)	166.0±33.4 (5)	138.4±33.6 (6)	0.466
**Ach (%)**	**pD_2_**	7.5±0.6 (9)	7.8±0.9 (8)	7.0±0.3 (7)	0.101
	**Maximal Response**	97.5 (71.7–100.1) (9)	90.6 (75.2–95.9) (8)	60.9 (55.6–80.1)^*^ (7)	**0.023**
**Ach + apocynin (%)**	**pD_2_**	7.2±1.0 (4)	8.7±0.4^*^ (5)	8.4±0.5^*^ (6)	**0.013**
	**Maximal Response**	101.1±1.4 (4)	96.4±5.3 (5)	94.8±6.6 (6)	0.213
**SNP (%)**	**pD_2_**	7.4±0.5 (6)	6.9±0.6 (5)	7.1±0.5 (5)	0.416
	**Maximal Response**	120.7±13.9 (6)	96.1±15.3^*^ (5)	92.3±6.5^*^ (5)	**0.005**
**SNP + apocynin (%)**	**pD_2_**	9.8±0.3 (4)	9.4±0.6 (5)	9.2±0.5 (5)	0.198
	**Maximal response**	102.2 (100.4–121.3) (4)	102.0 (100.0–105.2) (5)	105.0 (103.2–108.0) (5)	0.382

Data are expressed as mean ± standard deviation of mean or median with 25 and 75 percentiles; numbers in parentheses indicate the numbers of animals included in each experimental group. C: control group (no supplementation with vitamin D); VD3: supplemented with 3,000 IU VD/kg of chow; VD10: supplemented with 10,000 IU VD/kg of chow. pD_2_: indicates -log EC_50_ (the concentration of agonist producing half-maximal response); Phe: phenylephrine; E^+^: endothelium-intact vessels; E^−^: endothelium-denuded vessels; Ach: acetylcholine; SNP: sodium nitroprusside. * p<0.05 versus control group.

These results are also listed in Table 3. The Ach pD_2_ values were not different among the groups. However, the maximal relaxation induced by Ach was significantly lower in the VD10 group than in the control group. Apocynin increased the Ach pD_2_ values in both the supplemented groups compared to the control, and reversed the impaired Ach relaxation in the VD10 group. Similarly, the NPS pD_2_ values were not different among the groups, whereas the SNP maximal response was impaired in both the supplemented groups compared to the control group. The NAPDH inhibitor apocynin restored the NPS relaxation.

**Figure 1:**
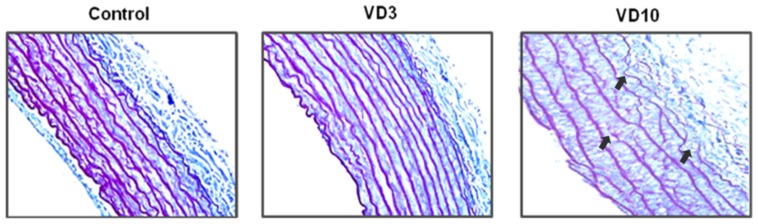
Elastin content in the aortic sections and fragmentation of elastic fibers in the VD10 group. Photographs of aortic samples (400×) stained by Calleja. C: control group (no supplementation with vitamin D); VD3: supplemented with 3,000 IU VD/kg of chow; VD10: supplemented with 10,000 IU VD/kg of chow.

Table 4 summarizes the results from histological analysis. The morphological data were not different when comparisons were performed among the three groups. However, the elastin volume fraction was lower, and the collagen volume fraction and the collagen/elastin ratio were higher in the VD10 group compared to the others groups. Moreover, the media of these animals (VD10) presented fragmentations of elastic fibers, which were observed in the arterial media of the samples from the VD10 group (Figure 1).

**Table 4:** Histological data of the aorta.

Variable	C	VD3	VD10	p
**CSA (μm^2^)**	7519±1555	6484±1129	7676±1642	0.370
	(6)	(6)	(3)	
**M (μm)**	13.3 (13.1–16.1)	12.0 (10.5–12.4)	12.9 (12.3–13.6)	0.235
	(6)	(6)	(3)	
**L (μm)**	157±14.9	155±13.3	175±28.1	0,269
	(6)	(6)	(3)	
**M/L**	0,087 (0,083–0,096)	0,072 (0,068–0,077)	0,070 (0,070–0,081)	0,130
	(6)	(6)	(3)	
**Collagen (%)**	0.26±0.03	0.22±0.03	0.29±0.03^#^	**<0.001**
	(8)	(8)	(8)	
**Elastin (%)**	0.35±0.04	0.35±0.03	0.30±0.03^*#^	**0.015**
	(9)	(8)	(9)	
**Collagen/elastin**	0.76±0.10	0.64±0.11	0.95±0.19 ^*#^	**<0.001**
	(8)	(8)	(7)	

Data are expressed as mean ± standard deviation of mean or median with 25 and 75 percentiles; numbers in parentheses indicate the numbers of animals included in each experimental group. C: control group (no supplementation with vitamin D); VD3: supplemented with 3,000 IU VD/kg of chow; VD10: supplemented with 10,000 IU VD/kg of chow. CSA: media cross-sectional area; M: media thickness; L; lumen diameter. * p<0.05 versus control group; # p<0.05 versus VD3 group.

Vascular mechanical properties are listed in Table 5. Supplementation with 3,000 IU of VD significantly had a lower aorta yield point compared to the control. No differences were observed among the groups for the other mechanical variables, failure load and stiffness.

**Table 5:** Data on the vascular mechanical properties and Metalloproteinase-2 and -9 activities by gelatin zimography.

Variable	C	VD3	VD10	P
**Yield point (N)**	4.2 (3.8–5.5) (10)	2.4 (2.1–2.9)^*^ (9)	3.8 (2.1–5.1) (9)	**0.009**
**Stiffness (N/mm)**	1.4±0.4 (10)	1.2±0.3 (9)	1.1±0.2 (9)	0.209
**Failure load (N)**	5.2±1.5 (10)	4.2±1.6 (9)	4.6±1.1 (9)	0.394
**Active/inactive MMP-2 (Arbitrary units)**	1.64 (0.72–2.06) (11)	1.19 (1.04–1.48) (11)	2.37 (1.49–3.76)^#^ (9)	**0.041**
**Active/inactive MMP-9 (Arbitrary units)**	5.90 (1.46–9.84) (10)	2.68 (1.69–5.02) (10)	4.33 (1.92–11.94) (9)	0.772

Data are expressed as mean ± standard deviation of mean or median with 25 and 75 percentiles, numbers in parentheses indicate the numbers of animals included in each experimental group. C: control group (no supplementation with vitamin D); VD3: supplemented with 3,000 IU VD/kg of chow; VD10: supplemented with 10,000 IU VD/kg of chow. * p<0.05 versus control group; # p<0.05 versus VD3 group.

The data describing the MMP-2 and MMP-9 activity are listed in Table 5. The ratio for active/inactive MMP-2 was higher in the VD10 group compared to the VD3 group. The ratios for the active/inactive forms of MMP-9 were not different when comparisons were performed among the three groups.

## Discussion

This study showed that VD supplementation for two months in normotensive rats is associated with higher arterial systolic pressure in these animals. In addition, there was higher aortic contractility, impairment of aortic relaxation, higher production of ROS, changes in collagen and elastin content and impairment of mechanical properties in the supplemented groups. These changes may be part of some mechanisms involved in the higher blood pressures that were found in the animals supplemented with VD.

In our study the animals supplemented with VD presented higher plasma 25(OH)D3. These values were consistent with data shown in studies which supplemented similar doses of VD [43], [57]. The rats receiving both VD doses showed a slight increase in serum calcium but were still normocalcemic [43], [57], [58], [59]. In addition, levels of 25(OH)D3 and calcium are below the values considered capable of generating toxicity signs by VD, as shown by Shepard and Deluca [43].

Consistent with previous reports [36], [37], [38], our data showed that VD supplementation in normotensive rats is associated with higher blood pressure. The elevation on the blood pressure was not associated with cardiac output nor with serum calcium in this model.

Over the past 2 decades, it has become apparent that VD is a modulator of vascular function [17]. Some VD actions are related to the increased vascular contractility [36], [60], [61], [62]. In our study, we found that VD supplementation led to an increased contractile response to Phe in aortas with denuded endothelium. These data are in accordance with other studies that demonstrated that 1,25-dihydroxyvitamin D increased the contractile force-generating capacity of the aorta and mesenteric arteries in both normotensive and hypertensive rats [36], [60], [62]. When the experiment was performed with endothelium, the contractile response returned to normal. We hypothesize that the endothelium possesses relaxation factors that controls the VD vasoconstriction effect. Therefore, this vasoconstriction effect alone does not explain the blood pressure elevation.

In addition, the animals supplemented with VD had impaired relaxation to both Ach and SNP. These results suggest the NO pathway or bioavailability could be impaired once Ach releases NO by the endothelial cell, while the SNP provides an inorganic source of NO [63], [64].

Superoxide anions (O_2_^−^) are largely responsible for altering the bioavailability of NO by forming peroxynitrite (ONOO^−^) [65]. Several enzymatic sources in blood vessels may produce ROS. NADPH oxidase complex is one of the most important of these sources and may be the largest producer of O_2_^−^ in the vascular wall [66]. Thus, we performed additional experiments in vessels in presence of NADPH oxidase inhibitor (apocynin). When the NADPH oxidase was inhibited by pre-incubation with apocynin, the vasorelaxation of supplemented animals improved and returned to similar vascular response to control group. Therefore, VD supplementation could be the responsible for the increased source of the ROS by NADPH oxidase complex and decreasing the NO bioavailability. This decreased NO bioavailability leads to impaired vascular relaxation, which may be a mechanism of increased arterial systolic pressure in this model.

Increased arterial pressure is also associated with structural and mechanical alterations in both resistance and conduit arteries [67]. The maintenance proper structural geometry, mechanical properties and function of vessels are dependent on the balanced composition of the extracellular components matrix (ECM) [68], [69]. We showed that supplemented animals did not present alterations in media cross-sectional area, media thickness and lumen diameter. However, they showed alterations in ECM. The collagen content was higher, and the elastin content was lower in the animals supplemented with highest VD dose. Therefore, the ratio collagen/elastin was elevated in these animals. However, no difference was observed in elasticity (yield point) and vascular stiffness. It can be speculated that occurred production of other collagen type or architecture rearranged in order to preserve the integrity and the mechanical properties of the vessel wall [70], [71].

The ECM metabolism is regulated for metalloproteinases (MMPs), which are Zn21- and Ca21-dependent proteolytic enzymes [72]. Several different MMPs are present in the vasculature. These MMPs include MMP-2 and MMP-9, which play an important role in vascular remodeling [73], [74], [75]. In our study, MMP2 activity was higher in the supplemented animals compared to control. Increased MMP-2 activity is associated with increased deposition of collagen, alterations in ECM architecture or ECM attachments [76], systemic arterial stiffness [77] and modulation of vascular contractility and relaxation [78], [79], thereby promoting vasoconstriction. Furthermore, MMP-2 activities are also associated with the destruction of the elastic lamina of arteries [80]. In situ studies showed gelatinolytic activity in tissue sections and strong MMP-2 immunostaining along the inner elastic lamina up to the lamina break [81]. Clinical and experimental studies have reported increased expression and activity of MMPs, particularly MMP-2 in the vascular tissues in animal hypertension models [51], [82]. Therefore, the alterations observed in ECM of supplemented animals may be associated with alterations in MMPs.

Studies have shown that increased oxidative stress and reduction in NO bioavailability both contribute to increased MMP-mediated vascular remodeling and resulting vascular pathologies [69], [83], [84]. In addition, during this process, ONOO^−^generated activates latent MMPs. These processes lead to the degradation of ECM components elastin and collagen. However, because the turnover of collagen is fast, more collagen is placed on the outer interstitial and inner medial layers of the aorta wall [84]. This placement may explain the relation between the higher collagen content observed in the VD10 group, which also had increased MMP-2 activity.

The higher collagen content in the group VD10 may also have prevented the increase in aortic contractility in these animals. Study has shown that the increased bulk collagen interposed between the smooth muscle cells reduced the force generation by the smooth muscle cells. Another possibility is that the attachments between smooth muscle cells and extracellular matrix are altered and influence the maximal tension generated [85]. Finally, VD supplementation in normotensive rats led to increased systolic blood pressure, but the mechanisms involved may be different, depending on the dose used. In the VD10 group, the dose was related to impaired vascular relaxation and changes in ECM. While in the VD3 group, the dose was related to increased vascular contractility and alterations of the aortic mechanical properties.

Several studies showed that VD exerts a biphasic “dose response” curve on cardiovascular physiopathology with deleterious consequences not only of VD deficiency but also of VD excess [86], [87]. Both VD deficiency [88], [89], [90], [91], [92], [93], [94] and high doses of VD [95], [96], [97], [98] can lead to structure and functional vascular alterations and hypertension. In addition, the VD deficiency is associated with marked increase in renin activity [99]. On the other hand, in hypertension models the VD presented antihypertensive effect. The antihypertensive mechanisms include the negative regulator for rennin, protects the vascular function and the inhibition of vascular smooth muscular cell proliferation and growth [31], [99], [100], [101], [102], [103].

In conclusion, our data suggest that the higher arterial pressure in normotensive rats after VD supplementation were caused by aortic alterations in function and structure. NO bioavailability and ROS production may also play an important role in this increased pressure.

## References

[1] Calvo MS, Whiting SJ, Barton CN (2005) Vitamin D intake: a global perspective of current status. J Nutr 135: 310–316. 15671233 10.1093/jn/135.2.310

[2] Holick MF (2007) Vitamin D deficiency. N Engl J Med 357: 266–281. 17634462 10.1056/NEJMra070553

[3] James WP (2008) 22nd Marabou Symposium: the changing faces of vitamin D. Nutr Rev 66: 286–290. 18454815 10.1111/j.1753-4887.2008.00034.x

[4] Kimball S, Fuleihan Gel H, Vieth R (2008) Vitamin D: a growing perspective. Crit Rev Clin Lab Sci 45: 339–414. 18568854 10.1080/10408360802165295

[5] Pittas AG, Lau J, Hu FB, Dawson-Hughes B (2007) The role of vitamin D and calcium in type 2 diabetes. A systematic review and meta-analysis. J Clin Endocrinol Metab 92: 2017–2029. 17389701 10.1210/jc.2007-0298 PMC2085234

[6] Hewison M (2008) Vitamin D and innate immunity. Curr Opin Investig Drugs 9: 485–490. 18465658

[7] Assalin HB, Rafacho BP, Santos PP, Ardisson LP, Roscani MG, et al (2013) Impact of the length of vitamin d deficiency on cardiac remodeling. Circ Heart Fail 6: 809–816. 23709660 10.1161/CIRCHEARTFAILURE.112.000298

[8] Weng S, Sprague JE, Oh J, Riek AE, Chin K, et al (2013) Vitamin D deficiency induces high blood pressure and accelerates atherosclerosis in mice. PLoS One 8: e54625. 23349943 10.1371/journal.pone.0054625 PMC3551761

[9] Seker T, Gur M, Kuloglu O, Kalkan GY, Sahin DY, et al (2013) Serum 25-hydroxyvitamin D is associated with both arterial and ventricular stiffness in healthy subjects. J Cardiol 62: 361–365. 23867332 10.1016/j.jjcc.2013.06.004

[10] Forman JP, Scott JB, Ng K, Drake BF, Suarez EG, et al (2013) Effect of vitamin D supplementation on blood pressure in blacks. Hypertension 61: 779–785. 23487599 10.1161/HYPERTENSIONAHA.111.00659 PMC3775458

[11] Andrukhova O, Slavic S, Zeitz U, Riesen SC, Heppelmann MS, et al (2014) Vitamin D is a regulator of endothelial nitric oxide synthase and arterial stiffness in mice. Mol Endocrinol 28: 53–64. 24284821 10.1210/me.2013-1252 PMC5426652

[12] Saraiva GL, Cendoroglo MS, Ramos LR, Araujo LM, Vieira JG, et al (2007) [Prevalence of vitamin D deficiency, insufficiency and secondary hyperparathyroidism in the elderly inpatients and living in the community of the city of Sao Paulo, Brazil]. Arq Bras Endocrinol Metabol 51: 437–442. 17546243 10.1590/s0004-27302007000300012

[13] Peters BS, dos Santos LC, Fisberg M, Wood RJ, Martini LA (2009) Prevalence of vitamin D insufficiency in Brazilian adolescents. Ann Nutr Metab 54: 15–21. 10.1159/000199454 19194104

[14] Pilz S, Tomaschitz A (2011) Vitamin D status: to be considered in heart failure patients!. Eur J Heart Fail 13: 595–596. 21613423 10.1093/eurjhf/hfr018

[15] Rosen CJ (2011) Clinical practice. Vitamin D insufficiency. N Engl J Med 364: 248–254. 21247315 10.1056/NEJMcp1009570

[16] IOM (2011) (Institute of Medicine). Dietary Reference Intakes for Calcium and Vitamin D. Washington, DC: The National academies Press. 1133 p. 21796828

[17] Bukoski RD, Kremer D (1991) Calcium-regulating hormones in hypertension: vascular actions. Am J Clin Nutr 54: 220S–226S. 2053566 10.1093/ajcn/54.1.220S

[18] Zehnder D, Bland R, Chana RS, Wheeler DC, Howie AJ, et al (2002) Synthesis of 1,25-dihydroxyvitamin D(3) by human endothelial cells is regulated by inflammatory cytokines: A novel autocrine determinant of vascular cell adhesion. Journal of the American Society of Nephrology 13: 621–629. 11856765 10.1681/ASN.V133621

[19] Somjen D, Weisman Y, Kohen F, Gayer B, Limor R, et al (2005) 25-hydroxyvitamin D3-1alpha-hydroxylase is expressed in human vascular smooth muscle cells and is upregulated by parathyroid hormone and estrogenic compounds. Circulation 111: 1666–1671. 15795327 10.1161/01.CIR.0000160353.27927.70

[20] Li YC (2011) Molecular mechanism of vitamin D in the cardiovascular system. J Investig Med 59: 868–871. 10.231/JIM.0b013e31820ee448 PMC3645939 21307778

[21] Bouillon R, Carmeliet G, Verlinden L, van Etten E, Verstuyf A, et al (2008) Vitamin D and human health: lessons from vitamin D receptor null mice. Endocr Rev 29: 726–776. 18694980 10.1210/er.2008-0004 PMC2583388

[22] Ishibashi K, Evans A, Shingu T, Bian K, Bukoski RD (1995) Differential expression and effect of 1,25-dihydroxyvitamin D3 on myosin in arterial tree of rats. Am J Physiol 269: C443–450. 7653526 10.1152/ajpcell.1995.269.2.C443

[23] Bian K, Ishibashi K, Bukoski RD (1996) 1,25(OH)2D3 modulates intracellular Ca2+ and force generation in resistance arteries. Am J Physiol 270: H230–237. 8769756 10.1152/ajpheart.1996.270.1.H230

[24] Rebsamen MC, Sun J, Norman AW, Liao JK (2002) 1alpha,25-dihydroxyvitamin D3 induces vascular smooth muscle cell migration via activation of phosphatidylinositol 3-kinase. Circ Res 91: 17–24. 12114317 10.1161/01.res.0000025269.60668.0f

[25] Tukaj C (2008) Enhanced proliferation of aortal smooth muscle cells treated by 1,25(OH)2D3 in vitro coincides with impaired formation of elastic fibres. Int J Exp Pathol 89: 117–124. 18336529 10.1111/j.1365-2613.2008.00578.x PMC2525765

[26] Pilz S, Tomaschitz A, Ritz E, Pieber TR (2009) Vitamin D status and arterial hypertension: a systematic review. Nat Rev Cardiol 6: 621–630. 19687790 10.1038/nrcardio.2009.135

[27] Witham MD, Nadir MA, Struthers AD (2009) Effect of vitamin D on blood pressure: a systematic review and meta-analysis. J Hypertens 27: 1948–1954. 19587609 10.1097/HJH.0b013e32832f075b

[28] Wu SH, Ho SC, Zhong L (2010) Effects of vitamin D supplementation on blood pressure. South Med J 103: 729–737. 20622727 10.1097/SMJ.0b013e3181e6d389

[29] Elamin MB, Abu Elnour NO, Elamin KB, Fatourechi MM, Alkatib AA, et al (2011) Vitamin D and cardiovascular outcomes: a systematic review and meta-analysis. J Clin Endocrinol Metab 96: 1931–1942. 21677037 10.1210/jc.2011-0398

[30] Margolis KL, Ray RM, Van Horn L, Manson JE, Allison MA, et al (2008) Effect of calcium and vitamin D supplementation on blood pressure: the Women's Health Initiative Randomized Trial. Hypertension 52: 847–855. 18824662 10.1161/HYPERTENSIONAHA.108.114991 PMC2791957

[31] Pfeifer M, Begerow B, Minne HW, Nachtigall D, Hansen C (2001) Effects of a short-term vitamin D(3) and calcium supplementation on blood pressure and parathyroid hormone levels in elderly women. J Clin Endocrinol Metab 86: 1633–1637. 11297596 10.1210/jcem.86.4.7393

[32] Wood AD, Secombes KR, Thies F, Aucott L, Black AJ, et al (2012) Vitamin D3 supplementation has no effect on conventional cardiovascular risk factors: a parallel-group, double-blind, placebo-controlled RCT. J Clin Endocrinol Metab 97: 3557–3568. 22865902 10.1210/jc.2012-2126

[33] Scragg R, Khaw KT, Murphy S (1995) Effect of winter oral vitamin D3 supplementation on cardiovascular risk factors in elderly adults. Eur J Clin Nutr 49: 640–646. 7498100

[34] Orwoll ES, Oviatt S (1990) Relationship of mineral metabolism and long-term calcium and cholecalciferol supplementation to blood pressure in normotensive men. Am J Clin Nutr 52: 717–721. 2169703 10.1093/ajcn/52.4.717

[35] Kienreich K, Grubler M, Tomaschitz A, Schmid J, Verheyen N, et al (2013) Vitamin D, arterial hypertension & cerebrovascular disease. Indian J Med Res 137: 669–679. 23703334 PMC3724247

[36] Bukoski RD, Xue H (1993) On the vascular inotropic action of 1,25-(OH)2 vitamin D3. Am J Hypertens 6: 388–396. 8512663 10.1093/ajh/6.5.388

[37] Haffner D, Hocher B, Muller D, Simon K, Konig K, et al (2005) Systemic cardiovascular disease in uremic rats induced by 1,25(OH)2D3. J Hypertens 23: 1067–1075. 15834294 10.1097/01.hjh.0000166849.72721.1c

[38] Santos PP, Assalin HB, Rafacho BP, Minicucci MF, Azevedo PS, et al (2011) Influence of different vitamin D doses on structure, function, energy metabolism and inflammatory mediators in the heart of Wistar rats. Eur Heart J 32: 719–719.

[39] Cowley AW Jr (1992) Long-term control of arterial blood pressure. Physiol Rev 72: 231–300. 1731371 10.1152/physrev.1992.72.1.231

[40] Oparil S, Zaman MA, Calhoun DA (2003) Pathogenesis of hypertension. Ann Intern Med 139: 761–776. 14597461 10.7326/0003-4819-139-9-200311040-00011

[41] Frohlich ED, Apstein C, Chobanian AV, Devereux RB, Dustan HP, et al (1992) The heart in hypertension. N Engl J Med 327: 998–1008. 1518549 10.1056/NEJM199210013271406

[42] Hrubec Z, Neel JV (1978) The National Academy of Sciences—National Research Council Twin Registry: ten years of operation. Prog Clin Biol Res 24 Pt B: 153–172. 569304

[43] Shepard RM, Deluca HF (1980) Plasma Concentrations of Vitamin D3 and Its Metabolites in the Rat as Influenced by Vitamin D, or 25-Hydroxyvitamin D3 Intake. Arch Biochem Biophys 202: 43–53. 6249223 10.1016/0003-9861(80)90404-x

[44] Pfeffer JM, Pfeffer MA, Frohlich ED (1971) Validity of an indirect tail-cuff method for determining systolic arterial pressure in unanesthetized normotensive and spontaneously hypertensive rats. J Lab Clin Med 78: 957–962. 5131859

[45] de Paiva SA, Zornoff LA, Okoshi MP, Okoshi K, Matsubara LS, et al (2003) Ventricular remodeling induced by retinoic acid supplementation in adult rats. Am J Physiol Heart Circ Physiol 284: H2242–2246. 12574000 10.1152/ajpheart.00646.2002

[46] Lang RM, Bierig M, Devereux RB, Flachskampf FA, Foster E, et al (2005) Recommendations for chamber quantification: a report from the American Society of Echocardiography's Guidelines and Standards Committee and the Chamber Quantification Writing Group, developed in conjunction with the European Association of Echocardiography, a branch of the European Society of Cardiology. J Am Soc Echocardiogr 18: 1440–1463. 16376782 10.1016/j.echo.2005.10.005

[47] Choi H, Allahdadi KJ, Tostes RC, Webb RC (2011) Augmented S-nitrosylation contributes to impaired relaxation in angiotensin II hypertensive mouse aorta: role of thioredoxin reductase. J Hypertens 29: 2359–2368. 22025239 10.1097/HJH.0b013e32834d2554 PMC4004364

[48] Castro MM, Rizzi E, Rascado RR, Nagassaki S, Bendhack LM, et al (2004) Atorvastatin enhances sildenafil-induced vasodilation through nitric oxide-mediated mechanisms. Eur J Pharmacol 498: 189–194. 15363994 10.1016/j.ejphar.2004.07.051

[49] Aksnes L (1992) A simplified high-performance liquid chromatographic method for determination of vitamin D3, 25-hydroxyvitamin D2 and 25-hydroxyvitamin D3 in human serum. Scand J Clin Lab Invest 52: 177–182. 1329183 10.3109/00365519209088782

[50] Dao HH, Lemay J, de Champlain J, deBlois D, Moreau P (2001) Norepinephrine-induced aortic hyperplasia and extracellular matrix deposition are endothelin-dependent. J Hypertens 19: 1965–1973. 11677361 10.1097/00004872-200111000-00006

[51] Castro MM, Rizzi E, Figueiredo-Lopes L, Fernandes K, Bendhack LM, et al (2008) Metalloproteinase inhibition ameliorates hypertension and prevents vascular dysfunction and remodeling in renovascular hypertensive rats. Atherosclerosis 198: 320–331. 18054360 10.1016/j.atherosclerosis.2007.10.011

[52] Cerqueira NF, Yoshida WB, Muller SS, Sequeira JL, de Rodrigues AC, et al (2005) Morphological and biomechanical study of abdominal aorta of rats submitted to experimental chronic alcoholism. Acta Cir Bras 20: 213–218. 16033179 10.1590/s0102-86502005000300004

[53] Castardeli E, Duarte DR, Minicucci MF, Azevedo PS, Matsubara BB, et al (2007) Tobacco smoke-induced left ventricular remodelling is not associated with metalloproteinase-2 or -9 activation. Eur J Heart Fail 9: 1081–1085. 17921050 10.1016/j.ejheart.2007.09.004

[54] Ardisson LP, Minicucci MF, Azevedo PS, Chiuso-Minicucci F, Matsubara BB, et al (2012) Influence of AIN-93 diet on mortality and cardiac remodeling after myocardial infarction in rats. Int J Cardiol 156: 265–269. 21095625 10.1016/j.ijcard.2010.10.128

[55] Cau SB, Guimaraes DA, Rizzi E, Ceron CS, Souza LL, et al (2011) Pyrrolidine dithiocarbamate down-regulates vascular matrix metalloproteinases and ameliorates vascular dysfunction and remodelling in renovascular hypertension. Br J Pharmacol 164: 372–381. 21434884 10.1111/j.1476-5381.2011.01360.x PMC3174417

[56] Seipelt RG, Backer CL, Mavroudis C, Stellmach V, Cornwell M, et al (2005) Local delivery of osteopontin attenuates vascular remodeling by altering matrix metalloproteinase-2 in a rabbit model of aortic injury. J Thorac Cardiovasc Surg 130: 355–362. 16077399 10.1016/j.jtcvs.2004.12.040

[57] Weishaar RE, Simpson RU (1987) Vitamin D3 and cardiovascular function in rats. J Clin Invest 79: 1706–1712. 3034981 10.1172/JCI113010 PMC424512

[58] O′Connell TD, Giacherio DA, Jarvis AK, Simpson RU (1995) Inhibition of cardiac myocyte maturation by 1,25-dihydroxyvitamin D3. Endocrinology 136: 482–488. 7835280 10.1210/endo.136.2.7835280

[59] Cardus A, Panizo S, Parisi E, Fernandez E, Valdivielso JM (2007) Differential effects of vitamin D analogs on vascular calcification. J Bone Miner Res 22: 860–866. 17352647 10.1359/jbmr.070305

[60] Bukoski RD, Wang DB, Wagman DW (1990) Injection of 1,25-(OH)2 vitamin D3 enhances resistance artery contractile properties. Hypertension 16: 523–531. 2228153 10.1161/01.hyp.16.5.523

[61] Bukoski RD, Li J, Bo J (1993) Effect of long-term administration of 1,25 (OH)2 vitamin D3 on blood pressure and resistance artery contractility in the spontaneously hypertensive rat. Am J Hypertens 6: 944–950. 8305169 10.1093/ajh/6.11.944

[62] Hatton DC, Xue H, DeMerritt JA, McCarron DA (1994) 1,25(OH)2 vitamin D3-induced alterations in vascular reactivity in the spontaneously hypertensive rat. Am J Med Sci 307 Suppl 1S154–158. 8141157

[63] Lind L (2008) Endothelium-dependent vasodilation in relation to different measurements of blood pressure in the elderly: the prospective investigation of the vasculature in Uppsala Seniors study. Blood Press Monit 13: 245–250. 18799948 10.1097/MBP.0b013e328305d286

[64] Li T, Wu HM, Wang F, Huang CQ, Yang M, et al.. (2011) Education programmes for people with diabetic kidney disease. Cochrane Database Syst Rev: CD007374. 10.1002/14651858.CD007374.pub2 21678365

[65] Fridovich I (1999) Fundamental aspects of reactive oxygen species, or what's the matter with oxygen? Ann N Y Acad Sci 893: 13–18. 10672226 10.1111/j.1749-6632.1999.tb07814.x

[66] Gorlach A, Brandes RP, Nguyen K, Amidi M, Dehghani F, et al (2000) A gp91phox containing NADPH oxidase selectively expressed in endothelial cells is a major source of oxygen radical generation in the arterial wall. Circ Res 87: 26–32. 10884368 10.1161/01.res.87.1.26

[67] Arribas SM, Hinek A, Gonzalez MC (2006) Elastic fibres and vascular structure in hypertension. Pharmacol Ther 111: 771–791. 16488477 10.1016/j.pharmthera.2005.12.003

[68] Bruel A, Oxlund H (1996) Changes in biomechanical properties, composition of collagen and elastin, and advanced glycation endproducts of the rat aorta in relation to age. Atherosclerosis 127: 155–165. 9125305 10.1016/s0021-9150(96)05947-3

[69] Tyagi SC (2000) Physiology and homeostasis of extracellular matrix: cardiovascular adaptation and remodeling. Pathophysiology 7: 177–182. 10996511 10.1016/s0928-4680(00)00046-8

[70] Bruel A, Ortoft G, Oxlund H (1998) Inhibition of cross-links in collagen is associated with reduced stiffness of the aorta in young rats. Atherosclerosis 140: 135–145. 9733224 10.1016/s0021-9150(98)00130-0

[71] Ponticos M, Smith BD (2014) Extracellular matrix synthesis in vascular disease: hypertension, and atherosclerosis. J Biomed Res 28: 25–39. 24474961 10.7555/JBR.27.20130064 PMC3904172

[72] Galis ZS, Sukhova GK, Lark MW, Libby P (1994) Increased expression of matrix metalloproteinases and matrix degrading activity in vulnerable regions of human atherosclerotic plaques. J Clin Invest 94: 2493–2503. 7989608 10.1172/JCI117619 PMC330083

[73] Bendeck MP, Zempo N, Clowes AW, Galardy RE, Reidy MA (1994) Smooth muscle cell migration and matrix metalloproteinase expression after arterial injury in the rat. Circ Res 75: 539–545. 8062427 10.1161/01.res.75.3.539

[74] Gibbons GH, Dzau VJ (1994) The emerging concept of vascular remodeling. N Engl J Med 330: 1431–1438. 8159199 10.1056/NEJM199405193302008

[75] Godin D, Ivan E, Johnson C, Magid R, Galis ZS (2000) Remodeling of carotid artery is associated with increased expression of matrix metalloproteinases in mouse blood flow cessation model. Circulation 102: 2861–2866. 11104745 10.1161/01.cir.102.23.2861

[76] Intengan HD, Schiffrin EL (2000) Structure and mechanical properties of resistance arteries in hypertension: role of adhesion molecules and extracellular matrix determinants. Hypertension 36: 312–318. 10988257 10.1161/01.hyp.36.3.312

[77] Medley TL, Cole TJ, Dart AM, Gatzka CD, Kingwell BA (2004) Matrix metalloproteinase-9 genotype influences large artery stiffness through effects on aortic gene and protein expression. Arterioscler Thromb Vasc Biol 24: 1479–1484. 15191941 10.1161/01.ATV.0000135656.49158.95

[78] Fernandez-Patron C, Stewart KG, Zhang Y, Koivunen E, Radomski MW, et al (2000) Vascular matrix metalloproteinase-2-dependent cleavage of calcitonin gene-related peptide promotes vasoconstriction. Circ Res 87: 670–676. 11029402 10.1161/01.res.87.8.670

[79] Martinez A, Oh HR, Unsworth EJ, Bregonzio C, Saavedra JM, et al (2004) Matrix metalloproteinase-2 cleavage of adrenomedullin produces a vasoconstrictor out of a vasodilator. Biochem J 383: 413–418. 15307819 10.1042/BJ20040920 PMC1133733

[80] Paik DC, Ramey WG, Dillon J, Tilson MD (1997) The nitrite/elastin reaction: implications for in vivo degenerative effects. Connect Tissue Res 36: 241–251. 9512892 10.3109/03008209709160224

[81] Lepetit H, Eddahibi S, Fadel E, Frisdal E, Munaut C, et al (2005) Smooth muscle cell matrix metalloproteinases in idiopathic pulmonary arterial hypertension. Eur Respir J 25: 834–842. 15863640 10.1183/09031936.05.00072504

[82] Bouvet C, Gilbert LA, Girardot D, deBlois D, Moreau P (2005) Different involvement of extracellular matrix components in small and large arteries during chronic NO synthase inhibition. Hypertension 45: 432–437. 15655118 10.1161/01.HYP.0000154680.44184.01

[83] Springman EB, Angleton EL, Birkedal-Hansen H, Van Wart HE (1990) Multiple modes of activation of latent human fibroblast collagenase: evidence for the role of a Cys73 active-site zinc complex in latency and a “cysteine switch” mechanism for activation. Proc Natl Acad Sci U S A 87: 364–368. 2153297 10.1073/pnas.87.1.364 PMC53264

[84] Steed MM, Tyagi N, Sen U, Schuschke DA, Joshua IG, et al (2010) Functional consequences of the collagen/elastin switch in vascular remodeling in hyperhomocysteinemic wild-type, eNOS-/-, and iNOS-/- mice. Am J Physiol Lung Cell Mol Physiol 299: L301–311. 20581102 10.1152/ajplung.00065.2010 PMC2951073

[85] Tozzi CA, Poiani GJ, Edelman NH, Riley DJ (1989) Vascular collagen affects reactivity of hypertensive pulmonary arteries of the rat. J Appl Physiol 66: 1730–1735. 2732163 10.1152/jappl.1989.66.4.1730

[86] Zittermann A, Schleithoff SS, Koerfer R (2007) Vitamin D and vascular calcification. Curr Opin Lipidol 18: 41–46. 17218831 10.1097/MOL.0b013e328011c6fc

[87] Dror Y, Giveon SM, Hoshen M, Feldhamer I, Balicer RD, et al (2013) Vitamin D levels for preventing acute coronary syndrome and mortality: evidence of a nonlinear association. J Clin Endocrinol Metab 98: 2160–2167. 23533239 10.1210/jc.2013-1185

[88] Watson KE, Abrolat ML, Malone LL, Hoeg JM, Doherty T, et al (1997) Active serum vitamin D levels are inversely correlated with coronary calcification. Circulation 96: 1755–1760. 9323058 10.1161/01.cir.96.6.1755

[89] Timms PM, Mannan N, Hitman GA, Noonan K, Mills PG, et al (2002) Circulating MMP9, vitamin D and variation in the TIMP-1 response with VDR genotype: mechanisms for inflammatory damage in chronic disorders? QJM 95: 787–796. 12454321 10.1093/qjmed/95.12.787

[90] Al Mheid I, Patel R, Murrow J, Morris A, Rahman A, et al (2011) Vitamin D status is associated with arterial stiffness and vascular dysfunction in healthy humans. J Am Coll Cardiol 58: 186–192. 21718915 10.1016/j.jacc.2011.02.051 PMC3896949

[91] Wasse H, Cardarelli F, De Staercke C, Hooper C, Veledar E, et al (2011) 25-hydroxyvitamin D concentration is inversely associated with serum MMP-9 in a cross-sectional study of African American ESRD patients. BMC Nephrol 12: 24. 21600051 10.1186/1471-2369-12-24 PMC3118225

[92] Giallauria F, Milaneschi Y, Tanaka T, Maggio M, Canepa M, et al (2012) Arterial stiffness and vitamin D levels: the Baltimore longitudinal study of aging. J Clin Endocrinol Metab 97: 3717–3723. 22767638 10.1210/jc.2012-1584 PMC3674293

[93] van de Luijtgaarden KM, Voute MT, Hoeks SE, Bakker EJ, Chonchol M, et al (2012) Vitamin D deficiency may be an independent risk factor for arterial disease. Eur J Vasc Endovasc Surg 44: 301–306. 22841360 10.1016/j.ejvs.2012.06.017

[94] Wong YY, Flicker L, Yeap BB, McCaul KA, Hankey GJ, et al (2013) Is hypovitaminosis D associated with abdominal aortic aneurysm, and is there a dose-response relationship? Eur J Vasc Endovasc Surg 45: 657–664. 23602862 10.1016/j.ejvs.2013.03.015

[95] Hinek A, Botney MD, Mecham RP, Parks WC (1991) Inhibition of tropoelastin expression by 1,25-dihydroxyvitamin D3. Connect Tissue Res 26: 155–166. 1769236 10.3109/03008209109152434

[96] Niederhoffer N, Lartaud-Idjouadiene I, Giummelly P, Duvivier C, Peslin R, et al (1997) Calcification of medial elastic fibers and aortic elasticity. Hypertension 29: 999–1006. 9095090 10.1161/01.hyp.29.4.999

[97] Jono S, Nishizawa Y, Shioi A, Morii H (1998) 1,25-Dihydroxyvitamin D3 increases in vitro vascular calcification by modulating secretion of endogenous parathyroid hormone-related peptide. Circulation 98: 1302–1306. 9751679 10.1161/01.cir.98.13.1302

[98] Qin X, Corriere MA, Matrisian LM, Guzman RJ (2006) Matrix metalloproteinase inhibition attenuates aortic calcification. Arterioscler Thromb Vasc Biol 26: 1510–1516. 16690876 10.1161/01.ATV.0000225807.76419.a7

[99] Li YC, Kong J, Wei M, Chen ZF, Liu SQ, et al (2002) 1,25-Dihydroxyvitamin D(3) is a negative endocrine regulator of the renin-angiotensin system. J Clin Invest 110: 229–238. 12122115 10.1172/JCI15219 PMC151055

[100] Carthy EP, Yamashita W, Hsu A, Ooi BS (1989) 1,25-Dihydroxyvitamin D3 and rat vascular smooth muscle cell growth. Hypertension 13: 954–959. 2786849 10.1161/01.hyp.13.6.954

[101] Mitsuhashi T, Morris RC Jr, Ives HE (1991) 1,25-dihydroxyvitamin D3 modulates growth of vascular smooth muscle cells. J Clin Invest 87: 1889–1895. 1645744 10.1172/JCI115213 PMC296939

[102] Borges AC, Feres T, Vianna LM, Paiva TB (1999) Effect of cholecalciferol treatment on the relaxant responses of spontaneously hypertensive rat arteries to acetylcholine. Hypertension 34: 897–901. 10523381 10.1161/01.hyp.34.4.897

[103] Sugden JA, Davies JI, Witham MD, Morris AD, Struthers AD (2008) Vitamin D improves endothelial function in patients with Type 2 diabetes mellitus and low vitamin D levels. Diabet Med 25: 320–325. 18279409 10.1111/j.1464-5491.2007.02360.x

